# Parallel evolution of genes controlling mitonuclear balance in short‐lived annual fishes

**DOI:** 10.1111/acel.12577

**Published:** 2017-03-11

**Authors:** Arne Sahm, Martin Bens, Matthias Platzer, Alessandro Cellerino

**Affiliations:** ^1^ Leibniz Insitute on AgeingFritz‐Lipmann InstituteJena07745Germany; ^2^ Bio@SNSScuola Normale SuperiorePisa56124Italy

**Keywords:** evolution, gerontogenes, lifespan, longevity regulation, longevity gene, molecular biology of aging, mortality

## Abstract

The current molecular understanding of the aging process derives almost exclusively from the study of random or targeted single‐gene mutations in highly inbred laboratory species, mostly invertebrates. Little information is available as to the genetic mechanisms responsible for natural lifespan variation and the evolution of lifespan, especially in vertebrates. Here, we investigated the pattern of positive selection in annual (i.e., short‐lived) and nonannual (i.e., longer‐lived) African killifishes to identify a genomic substrate for evolution of annual life history (and reduced lifespan). We identified genes under positive selection in all steps of mitochondrial biogenesis: mitochondrial (mt) DNA replication, transcription from mt promoters, processing and stabilization of mt RNAs, mt translation, assembly of respiratory chain complexes, and electron transport chain. Signs of paralleled evolution (i.e., evolution in more than one branch of *Nothobranchius* phylogeny) are observed in four out of five steps. Moreover, some genes under positive selection in *Nothobranchius* are under positive selection also in long‐lived mammals such as bats and mole‐rats. Complexes of the respiratory chain are formed in a coordinates multistep process where nuclearly and mitochondrially encoded components are assembled and inserted into the inner mitochondrial membrane. The coordination of this process is named mitonuclear balance, and experimental manipulations of mitonuclear balance can increase longevity of laboratory species. Our data strongly indicate that these genes are also casually linked to evolution lifespan in vertebrates.

## Introduction

The current molecular understanding of the aging process derives almost exclusively from the study of random or targeted single‐gene mutations in highly inbred laboratory species, mostly invertebrates. Little information is available as to the genetic mechanisms responsible for natural lifespan variation and the evolution of longevity, especially in vertebrates. Yet, natural variability in lifespan across vertebrate species greatly exceeds the magnitude of life extension that has been obtained by single‐gene manipulations, and a comparative approach may reveal novel genetic pathways that are responsible for evolution of lifespan.

The increasing availability of sequenced genomes and transcriptomes of related species with differing lifespans can facilitate the identification of putative aging‐related genes by analysis of positive selection. Positive selection is the evolutionary process by which a mutation becomes fixed in a population because it increases fitness. If two branches of an evolutionary tree differ in a key phenotype (lifespan, in this case), the genes under positive selection likely played a role in the evolution of that phenotype. In interspecies comparisons, positive selection on protein‐coding sequences results in an increase in the rate of non‐synonymous substitutions as compared with random genetic drift. Statistical models based on the ratio of non‐synonymous to synonymous substitution rates (*d*_*N*_/*d*_*S*_) can identify specific amino acid codons within a given gene that evolved due to positive selection and are widely used in comparative genomics (Kosiol *et al*., 2008; Roux *et al*., 2014; Davies *et al*., 2015).

One of the main limitations in applying this approach to the investigation of the genetic basis for lifespan evolution is the lack of a group of related species that are good laboratory organisms, are genetically tractable, and at the same time show naturally evolved large‐scale differences in lifespan. Genome‐wide scans for positive selection were performed in several long‐lived mammals (bats, the naked mole‐rat, the bowhead whale). However, it is not possible to establish a link between positively selected genes (PSGs) and evolution of longevity because the short‐lived sister *taxon* (i.e., the most closely related species/clade) may not be available for analysis, making it impossible to exclude that of a codon change was selected before longevity evolved [for a discussion see (Sahm *et al*., 2016a)] and it is very often impossible to relate a codon change to one of the several traits that distinguish two taxa (e.g., a PSG in *H. sapiens* may be related to longevity, bipedalism, absence of fur, speech, relative brain size, or any other trait that distinguish humans from apes).

Annual fishes of the genus *Nothobranchius* are small teleost fishes from East Africa adapted to the alternation of wet and rainy season. They inhabit ephemeral habitats that last a few months (Tozzini *et al*., 2013). This short lifespan is retained under captive conditions and is coupled to rapid expression of a host of conserved age‐associated phenotypes (Cellerino *et al*., 2016). In addition, a key adaptation of annual fishes is the ability to enter diapause – a state when development halts – at specific stages during embryonic life, that is necessary to survive the dry season. The genus *Nothobranchius* evolved from a non‐annual (therefore longer‐lived) ancestor, the non‐annual sister genus (Aphyosemion), is clearly identified (Furness *et al*., 2015), and the two taxa provide a sharp phenotypic contrast. Duration of the habitat (aridity) strictly limits natural lifespan of Nothobranchius fishes.

We specifically tested whether differences in habitat duration led to the evolution of a different rate of senescence in Nothobranchius populations from southern and central Mozambique, a region characterized by a major gradient in aridity. Two independent evolutionary lineages of Nothobranchius are found in this area: *N. furzeri* and *N. kuhntae* belong to one lineage while *N. rachovii* and *N. pienaari* belong to another lineage (Dorn *et al*., 2014). For each lineage, one species originates from semi‐arid habitat (*N. furzeri* and *N. pienaari*, respectively) and another species from the humid habitat (*N. kuhntae* and *N. rachovii*, respectively). In both species pairs, the species from more arid habitats showed shortened lifespan and accelerated expression of aging markers (Tozzini *et al*., 2013), thereby providing a clear example of parallel evolution.

We previously sequenced and assembled the genome of *N. furzeri* as well as the transcriptomes of *N. kadleci* (the sister species of *N. furzeri*), *N. pienaari*,* N. rachovii*, and *N. kuhntae* together with *N. korthause* [a long‐lived *Nothobranchius*, lifespan 18 months (Baumgart *et al*., 2015)], and *Aphyosemion striatum* (lifespan > 3 years) as a representative of the non‐annual sister genus (Reichwald *et al*., 2015). We found seven genes under positive selection in *N. furzeri* and one in *N. pienaari*, another very‐short‐lived species, using the other six species of Nothobranchiidae as outgroups (Reichwald *et al*., 2015). Here, we use a different selection of outgroups and analyze deeper branches of the *N. furzeri* phylogenetic tree to identify PSGs: (i) in coincidence with the evolution of annual life and (ii) showing parallel evolution in the two clades that are found in southern and central Mozambique.

Some results of this study were published in the form of preprint (Sahm *et al*., 2016b).

## Results

### Genomewide scan strategy

**Figure 1:**
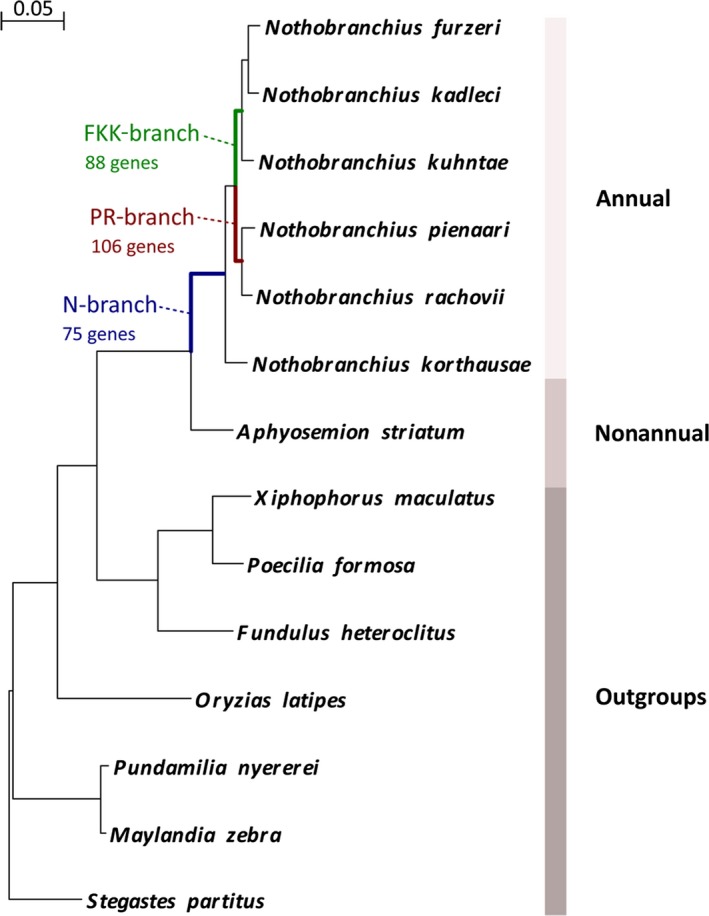
Phylogeny of the analyzed species. Maximum‐likelihood tree figure. Phylogeny of the analyzed species and their life history. Maximum‐likelihood nucleotide‐based phylogenetic tree of species that were used for genome‐scale scans for positively selected genes. Outgroups from Ovalentaria are indicated as well as the three branches (N‐, PR‐, and FKK‐branch) that are reported in the text. The alignment is based on concatenation of 4865 genes. The represented tree is the consensus of 1046 different trees created by splitting the alignment in fragments of 15 knt and calculating a tree for each fragment. The calibration bar refers to substitutions per nucleotidic site.

In addition to sequence data presented previously (Reichwald *et al*., 2015), we obtained from GenBank the RefSeq mRNA sequences of the phylogenetically closest outgroups from Ovalentaria (Fig. 1) and analyzed the pattern of positive selection along three internal branches of the tree: The first branch corresponds to the last common ancestor (LCA) of all *Nothobranchius* spp. (N‐branch) and it marks the transition to annual life cycle. The other two branches correspond to the LCA of *N. pienaari* and *N. rachovii* (PR‐branch) and LCA of *N. furzeri*,* N. kadleci* and *N. kuhntae* (FKK‐branch), respectively. These two branches diverged in the Pleistocene, share the same distribution, and species belonging to the two clades can be found sympatric in the same pond (Dorn *et al*., 2014). They represent therefore independent adaptations to the paleoclimatic changes of that period that was characterized by long‐term progressive aridification of East Africa (Dorn *et al*., 2014) and likely they were both subject to continued selection on adaptations linked to annual life cycle.

**Figure 2:**
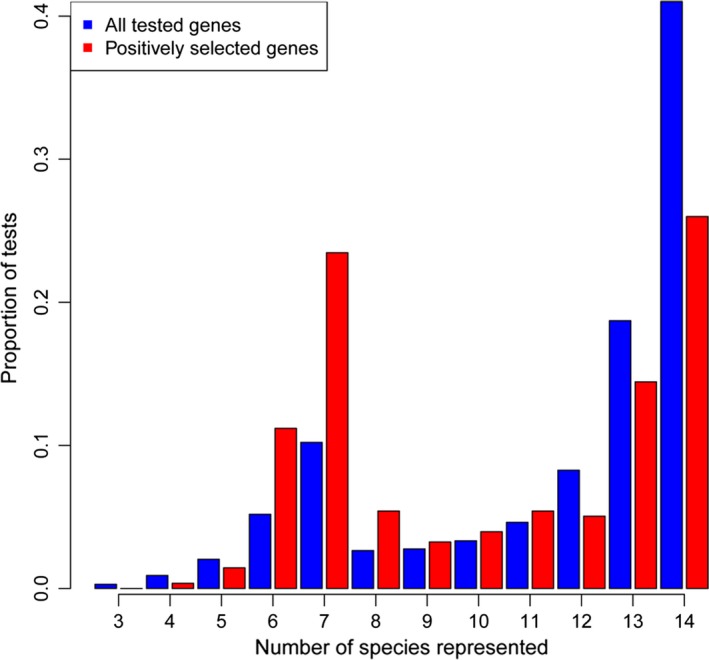
Distribution of taxon coverage for all tested genes (blue bars) and the positively selected genes (red bars). The *X*‐axis reports number of taxa for which a gene sequence is available, and the *Y*‐axis reports the fraction of genes falling into each coverage class.

In each calculation, the background was the union of all the branches of the tree excluding the respective foreground branch, that is, when studying the N‐branch the FKK and PR (and their child branches) are included in the background. In all comparisons, we defined PSGs based on nominal significant *P*‐values (i.e., < 0.05, not corrected for multiple testing). This was a deliberate choice because of several reasons. First, we aim primarily at identifying parallel evolution at the level of pathway and not individual genes. Second, the number of genes strongly influences the sensitivity of Fisher's exact test, and it is not meaningful to perform GO analysis on lists containing few genes. Third, *de novo* transcriptome assembly projects inevitably generate incomplete data and a fraction of genes will show incomplete taxon coverage. We specifically tested whether PSGs have higher taxon coverage than the whole set of tested genes. However, this was not the case (Fig. 2) and only one PSG has a taxon coverage smaller than five.

### Positive selection acts on mitochondrial and mitonuclear balance proteins

**Figure 3:**
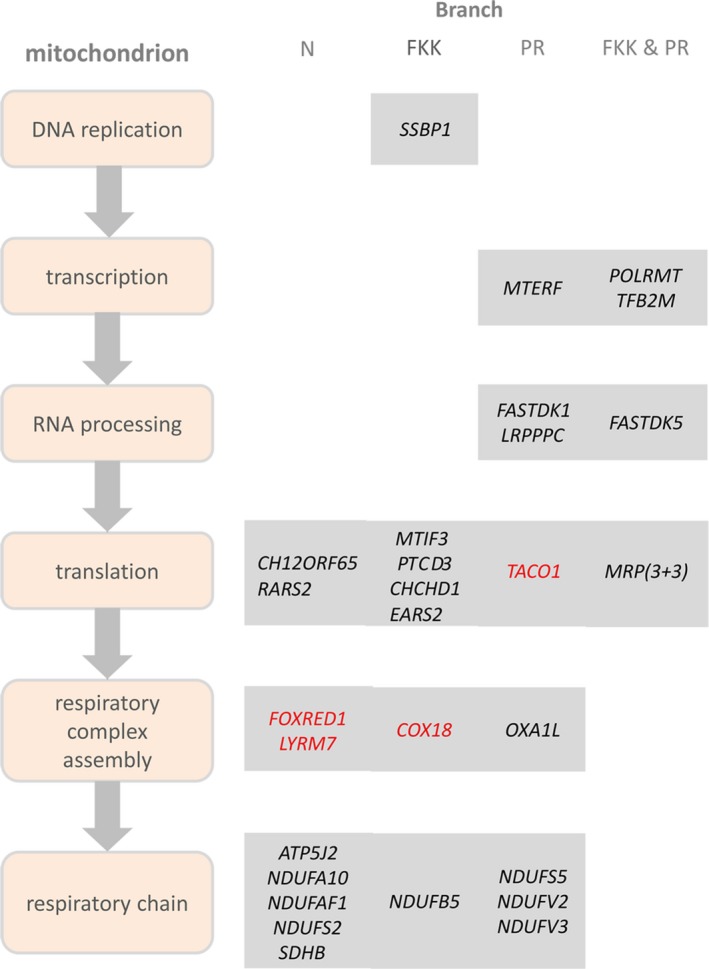
Genes controlling mitochondrial biogenesis and mitonuclear balance under selection in the three branches. Mitochondrial biogenesis was divided into the following processes: mtDNA replication, transcription from mitochondrial promoters, processing and stabilization of mitochondrial RNAs, translation, assembly of respiratory chain complexes and electron transport chain. Genes in black are classified based on their GO annotation genes in red genes are involved in mitochondrial biogenesis based on literature but not annotated as such in GO (see text for references). The term MRP indicates mitochondrial ribosomal proteins (MRPL53, MRPS31, and MPRS26 in FKK‐branch and MRPL23, MRPL3, and MTG2 in the RP‐branch, respectively).

We found 75 PSGs in the N‐branch, 106 in the PR‐branch, and 88 in the FKK‐branch (*P* < 0.05, branch‐site test; Tables S1–S3, Supporting information). Among these, four code for components of the mitochondrial respiratory chain complex I in the N‐branch (GO:0005747, fold‐enrichment = 14, *P* = 0.0002, Fisher's exact test; Fig. 3, Table S1, Supporting information). Therefore, emergence of annual life cycle is coincident with strong positive selection on mitochondrial respiration. This is in line with the evidence that diapause is linked to profound remodeling of mitochondrial physiology (Duerr & Podrabsky 2010). Three further genes of complex I are under positive selection in the PR‐branch (fold‐enrichment = 8.8, *P* = 0.005, Fisher's exact test) and one further gene in the FKK‐branch, indicating parallel and continued positive selection on complex I during the evolutionary history of Nothobranchius (Fig. 3, Tables S2 and S3, Supporting information).

Strikingly, other nine genes were under positive selection in both the PR‐ and FKK‐branches (Table 1). Among these, are *TFB2M* (transcription factor B2, mitochondrial) and *POLRMT* (polymerase (RNA) mitochondrial) that together with *TFAM* (transcription factor A, mitochondrial) form the ternary complex that transcribes the entire mitochondrial genome (Litonin *et al*. 2010) and *FASTKD5* (fast kinase domain 5) that is necessary for processing of mitochondrial mRNAs (Antonicka & Shoubridge 2015). Further signs of parallel positive selection were evident at the level of functional gene groups. In addition to *FASTKD5*,* FASTKD1* and *LRPPPC* (leucine‐rich pentatricopeptide repeat containing), that control stability of mitochondrial RNAs (Sasarman *et al*. 2010), were positively selected in PR‐branch. Three mitochondrial ribosome proteins (MRPs) were under positive selection in each of the two branches (GO:0005761, fold‐enrichment = 9.1 and 14.7, respectively, *P* = 0.02 and 0.01, Fisher's exact test for the PR‐ and FKK‐branch, respectively). In addition, two recently identified MRPs (Koc *et al*. 2013) were positively selected in FKK‐branch: *PTCD3* (pentatricopeptide repeat‐containing protein 3) and *CHCHD1* (coiled‐coil‐helix‐coiled‐coil‐helix domain containing protein 1). Two further genes important for translation of mitochondrial RNAs were also positively selected: *MTIF3* (mitochondrial translation initiation factor 3) in FKK‐branch and *TACO1* (translational activator of mitochondrially encoded cytochrome C oxidase I) in PR‐branch (Fig. 3).

**Table 1:** Genes that are positively selected both in PR‐ and FKK‐branch

Gene symbol	Gene name	Function
*ETAA1*	Ewing tumor‐associated antigen 1	DNA damage response
*POLRMT*	Polymerase (RNA) mitochondrial (DNA directed)	Transcription of mtDNA
*PRRC2C*	Proline‐rich coiled‐coil 2C	Poly‐A RNA binding
*APOA1*	Apolipoprotein A‐I	High‐density lipoprotein particle binding
*FASTKD5*	FAST kinase domains 5	Regulation of mitochondrial RNA stability
*TAF1C*	TATA box‐binding protein (TBP)‐associated factor, RNA polymerase I, C, 110 kDa	Transcription of nuclear DNA
*TFB2M*	Transcription factor B2, mitochondrial	Transcription of mtDNA
*CLIP1A* (Nfu_g_1_008997)	CAP‐GLY domain containing linker protein 1a	Unknown
*SI:DKEYP‐77H1.4 (Nfu_g_1_001190)*	Uncharacterized	

Respiratory chain complexes are large protein complexes that undergo multistep assembly where nuclearly and mitochondrially encoded components are combined and inserted into the mitochondrial inner membrane (Ghezzi & Zeviani 2012). Several genes involved in this process were positively selected: *COX18* (cytochrome C oxidase assembly factor) (Sacconi *et al*. 2009) in FKK‐branch, *OXA1*L (oxidase (cytochrome c) assembly 1‐like) (Stiburek *et al*. 2007; Haque *et al*. 2010) in PR‐branch, *FOXRED1* (FAD‐dependent oxidoreductase domain containing 1; Fassone *et al*. 2010) and *LYRM7* (LYR motif containing 7) (Sanchez *et al*. 2013) in N‐branch (Fig. 3). Therefore, proteins necessary for mitochondrial biogenesis and more specifically for expression of mitochondrially encoded genes and assembly of respiratory chain complexes show signs of parallel evolution. Altogether, among the observed 269 cases of positive selection along the three branches, 33 could be assigned to mitochondrial proteins and those involved in the mitochondrial biogenesis and mitonuclear balance (Fig. 3 and Tables S1–S3, Supporting information).

We also compared expression levels of genes in mitochondrial biogenesis and mitonuclear balance in two contrasts of a short‐ and a long‐lived species: *N. furzeri* vs. *A. striatum* and mouse vs. naked mole‐rat (Yu *et al*., 2011). For the genes, 1‐to‐1 orthology relationships based on ENSEMBL IDs could be established for 23. Of these, 12 (*RARS2*,* FASTKD5*,* POLRMT*,* OXA1L*,* NDUFAF1*,* C12orf65*,* NDUFS2*,* MTG2*,* PTCD3*,* MRPS31*,* NDUFS5*,* NDUFB5*) have a lower expression in both long‐lived species (*P*‐value = 0.005985, binomial test, ¼, Table S4, Supporting information).

### Gene enrichment is not due to expression bias or incomplete lineage sorting

**Figure 4:**
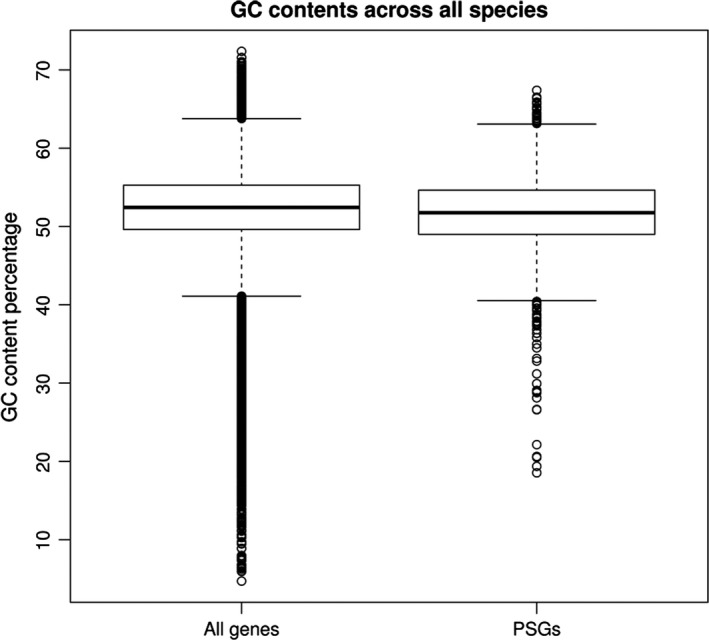
Distribution of GC content in all the tested transcripts and the positively selected genes. Data from all species and tests are pooled. Box plots indicate 10%, 25%, median, 75% and 90% quantiles, and points represent outliers.

To ensure the statistical significance of our observation and exclude that biases due to the transcriptome assembly process and sequencing biases – in particular toward highly expressed genes – are responsible for the enrichment of mitochondrial proteins, we performed a simulation where we built two gene sets for each of three tested branches: an expression‐adjusted background gene set and a “mitochondrial biogenesis” gene set. The later was derived from the union of the GO categories mitochondrial RNA metabolic process (GO:0000959), mitochondrial translation (GO:0032543), cellular respiration (GO: 0045333), mitochondrial respiratory chain complex assembly (GO:0033108), mitochondrial morphogenesis (GO:0070584). Per simulation run, we then randomly draw for each of the three branches from the background set a number of genes that equals the number of PSGs that were identified in the respective branch and calculated the sum of drawn mitochondrial biogenesis genes. In none of 1.000.000 simulation runs, a higher number than 21 was observed (95% quantile: 11). We concluded that our finding of 33 cases of positive selection on mitochondrial biogenesis genes is highly significant (simulated *P* < 10^−6^) and not caused by an expression or sequencing bias. Analysis of GC content demonstrated that PSGs did not differ from all analyzed genes (Fig. 4).

To exclude that enrichment was due to incomplete lineage sorting (Mendes & Hahn, 2016), we tested all PSGs that were identified initially based on a globally estimated tree again with a tree that was estimated using only the individually tested gene. Among all PSGs, 91% (244/269) were supported by this approach as well. Only one gene involved in mitochondrial biogenesis, namely *SDHB* in the N‐branch, was not confirmed to be a PSG by this analysis.

### Positively selected genes are enriched among annualism‐related genes

**Figure 5:**
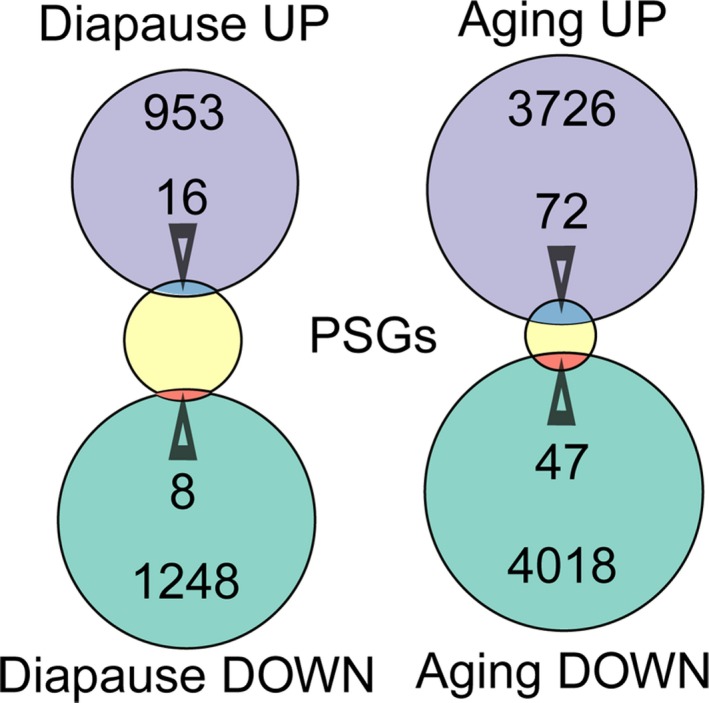
Overlap of positively selected genes (PSGs) with genes regulated during diapause or aging. Differentially expressed genes were obtained from Baumgart *et al*. (2014) and Reichwald *et al*. (2015) for brain aging and diapause, respectively. The arrowheads point to the intersection of the sets and indicate the number of genes in the respective intersections. The numbers within the circles indicate the number of genes in each set excluded from the intersections. The total number of PSGs in 267 in both cases.

To derive independent evidence that the PSGs may be involved in the evolution of annual life style, we compared the union of the PSGs with two sets of differentially expressed genes (DEGs) in *N. furzeri*: (i) DEGs detected during brain aging (Baumgart *et al*., 2014) and (ii) DEGs detected during diapause (Reichwald *et al*., 2015). PSGs showed an over‐representation among upregulated DEGs during diapause (*P* = 0.023, respectively, Fisher's exact test, Fig. 5) and among these 17 genes, *TFB2M* (PSG in both PR‐ and FKK‐branch) and the assembly factor *NDUFA1* (PSG in the N‐branch) are of relevance for mitonuclear balance. Over‐representation of PSGs among upregulated DEGs is observed also during aging (*P* = 0.0093, respectively, Fisher's exact test, Fig. 5), among these 47 genes, TFB2M is again present. PSGs upregulated during aging were also four genes of the cytokine–cytokine receptor interaction pathway (*CSF1RA*,* FLT1*,* IL2RGA*,* IL2ST*; dre04060 KEGG, *P* = 0.0001, Fisher's exact test).

**Figure 6:**
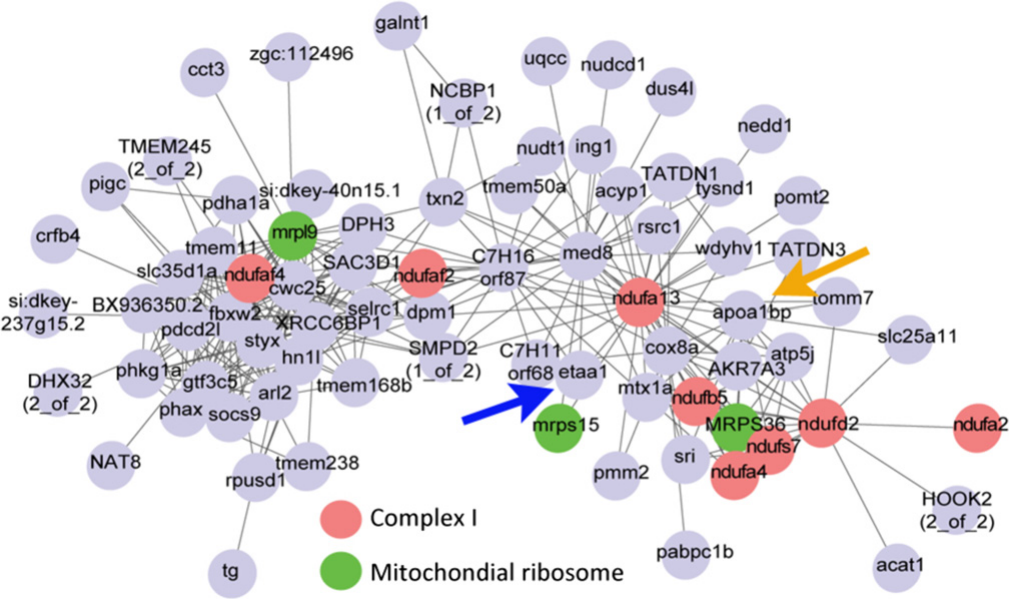
Position of ETAA1 and APOA1BP in the network of longevity‐associated genes described by Baumgart *et al*. (2016). Picture reproduced with permission from Baumgart *et al*. (2016) and the gene annotation conforms to the annotation of transcripts described in Reichwald *et al*. (2015). Red genes code for complex I components and green genes for mitochondrial ribosome components.

In addition, we compared PSGs with results of longitudinal gene expression in *N. furzeri*. Gene co‐expression network analysis revealed that *ETAA1*, positively selected in both PR‐ and FKK‐branch, and *APOA1BP* (apolipoprotein A1 binding protein), the binding partner of the PSG *APOA1,* are part of a module of co‐regulated genes highly enriched with MRPs and complex I components and negatively correlated with longevity (Baumgart *et al*., 2016; Fig. 6).

### Positively selected genes overlap with those in long‐lived mammals

Previous analysis of PSGs in the *N. furzeri* genome suggested that some aging‐relevant genes (e.g., the insulin like growth factor 1 receptor) can be positively selected both in short‐ and long‐lived species (Valenzano *et al*., 2015). We therefore compared *Nothobranchius* PSGs with PSGs detected by others in six species/clades of long‐lived mammals (naked mole‐rat, mole‐rat LCA, blind mole‐rat, human, bowhead whale and Brandt's bat). In all species, some PSGs overlap with those detected in *Nothobranchius* (Table S13, Supporting information). Of particular interest because under selection in more than two species/branches are: (i) *POLRMT*, that is a PSG in PR‐ and FKK‐branch as well as in the Brandt's bat and the two extracellular matrix genes (ii) tenascin (*TNC*), a PSG in humans, mole‐rats and in the FKK‐branch regulated during both aging and diapause and (iii) Collagen type IV alpha 2 (*COL4A2*), a PSG in the naked mole‐rat, the mole‐rat LCA and in the FKK‐branch also regulated during aging.

## Discussion

The coordinated synthesis and assembly of mitochondrially and nuclearly encoded components of the respiratory chain (mitonuclear balance) is a conserved longevity mechanism that is controlled by MRPs (Dillin *et al*., 2002; Houtkooper *et al*., 2013). Knock‐down of MRPs during early life in *C. elegans* results in an impaired assembly of respiratory complexes and life extension. Studies in the mouse and *N. furzeri* have shown that MRPs and nuclearly encoded complex I components are tightly co‐regulated and expression of these genes during early adult life is predictive of lifespan in vertebrates (Miwa *et al*., 2014; Baumgart *et al*., 2016). Further, inhibition of complex I activity during adult life prolongs lifespan and rejuvenates the transcriptome in *N. furzeri* (Baumgart *et al*., 2016).

Here, we show that these same genes are under positive selection in annual fish strongly suggesting that that evolution of the genes controlling mitonuclear balance is causally linked to evolution of short lifespan and annual life cycle. This is supported by our findings that several of these genes are positively selected along more than one investigated branch and are differentially regulated during diapause and aging of the shortest lived *Nothobranchius* species.

At the single‐gene level, of particular interest are PSGs that are detected both in the PR‐ and FKK‐branch that represent examples of parallel evolution. *POLRMT* and *TFB2M* are part of the ternary complex that transcribes mitochondrial DNA (including mitochondrial rRNAs) and *TAF1C* (TATA box‐binding protein‐associated factor RNA polymerase I subunit C) that is part of the multisubunit SL1 complex, which is required for RNA polymerase I to synthesize ribosomal RNA. Therefore, these three genes are at the core of the process that controls the balance between biogenesis of cytosolic and mitochondrial ribosomes. *APOA1* (apolipoprotein A1) is a component of HDL particles that have an obvious relevance for human age‐related diseases. Polymorphisms of *APOA1* are associated with coronary artery disease (Helgadottir *et al*., 2016) and it is an interactor of the *APOE*, a well‐described genetic risk factor for Alzheimer's and cardiovascular diseases (Mahley, 2016) and the locus with the largest statistical support for an association with extreme longevity (Broer *et al*., 2015). Interestingly, its expression in the liver is correlated with body weight in mice (Pearson's correlation coefficient: −0.84 for females and −0.74 for males, http://phenome.jax.org/). *EAAT1* shows striking similarities with *APOA1*. Its expression in the liver correlates with female body weight in mice (Pearson's correlation coefficient: +0.94). *ETAA1* and *APOA1BP* have central positions in the gene module of co‐expressed genes whose expression is negatively correlated with lifespan that also contains MRPs and complex I (Fig. 6), strongly suggesting that they are involved in mitonuclear balance. Interestingly, a function of *ETAA1* in DNA repair was recently demonstrated (Lee *et al*., 2016) and the gene coding for another protein important in DNA repair, *XRCC5*, was previously shown to be under positive selection in *N. furzeri* (Valenzano *et al*., 2015; Sahm *et al*., 2016a). However, it is not possible to determine *in silico* whether the substitutions observed in the two lineages cause similar changes of mitochondrial function and parallel selection on the same genes does not represent a proof of functional convergence.

Are the genes under selection in short‐lived species also involved in evolution of longevity? Data supporting this notion come from different studies of positive selection in the genomes of long‐lived species. Ant workers can live on average ten times as long as their solitary ancestors and queens with 10 years at average and nearly 30 years at maximum even more than 100 times as long (Jemielity *et al*., 2005). In an examination of seven ants genomes, highly significant enrichments of PSGs were documented for a series of GO terms that are related to the respiratory chain or mitochondrial biogenesis; especially mitochondrial electron transport (GO:0006120), mitochondrial respiratory chain complex I (GO:0005747), and mitochondrial large/small ribosomal subunit (GO:0005762/GO:0005763) (Roux *et al*., 2014). The same study reported based on expression data obtained in the fire ant *S. invicata* that PSGs are highly expressed in queens, intermediately expressed in workers and weakest expressed in males which are the shortest lived ant caste. While the expression of PSGs correlates with lifespan of the respective caste, there is no differential expression across mitochondrial genes in general between queens and workers. This means that the association between PSGs and caste biased gene expression cannot be simply explained by higher overall levels of genes that are involved in mitochondrial activity but suggests a relation between queen‐specific expression of PSGs and longer lifespan. Notably, consistent with the results of our study, there was no evidence found for positive selection on mitochondrial‐encoded genes in ants. Furthermore, respiratory chain genes were found to be under positive selection in the bats *P. poliocephalus* and *M. lucifugus* (Shen *et al*., 2010). Both are long‐lived mammals, while *P. poliocephalus* reaches a maximum age of 23.6 years at a weight of 675 g resulting in a lifespan that is 1.7 times larger than expected based on the body mass, *M. lucifugus* even reaches a maximum age of 34 at a weight of only 10 g resulting in lifespan almost five times longer than expected based on body mass (Tacutu *et al*., 2013).

Overlaps with long‐lived mammals are detectable also at the level of single genes. Of particular interest is *POLRMT* this gene that codes for the mitochondrial RNA polymerase is a PSG in the PR‐ and FKK‐branch and also in the Brandt's bat (Seim *et al*., 2013) and, as discussed above, it is of key importance for mitonuclear balance. It is tempting to speculate that positive selection in short‐ and long‐lived species modulates mitochondrial function in opposite directions. However, as discussed above, it is not possible to predict the functional impact of molecular evolution and this hypothesis will require experimental tests. Indirect evidence in favor comes from the observation that a significant fraction of mitochondrial biogenesis and mitonuclear balance genes are lower expressed in the longer lived element of two comparisons of long‐ and short‐lived species: *N. furzeri* vs. *A. striatum* and mouse vs. naked mole‐rat.

This hypothesis is also supported by direct measurements of complex I activity. Assays of mitochondrial physiology in the bivalve *Arctica islandica* (the longest lived metazoan with maximum lifespan exceeding 500 years) and two taxonomically related species of comparable size revealed lower activity of complex I resulting in reduced production of reactive oxygen species (Munro *et al*., 2013). Similarly, low activity of complex I and low production of reactive oxygen species were related to longevity in homeotherm vertebrates (Brunet‐Rossinni, 2004; Lambert *et al*., 2010) and, finally, conditions that increase mouse longevity are associated with reduced expression of complex I (Miwa *et al*., 2014).

Comparison of positive selection at the gene level between *Nothobranchius* and long‐living mammals identified *TNC* and *COL4A2* as particularly interesting candidates as they are PSGs in two mammalian clades each and are also both differentially expressed in *Nothobranchius furzeri* aging (Reichwald *et al*., 2015). These data lend further support to the notion that extracellular matrix genes are regulators of lifespan that derives from meta‐analysis of genomewide transcript regulation (de Magalhaes *et al*. 2009), positive selection analysis (Li & de Magalhaes, 2013), and experimental approaches (Ewald *et al*., 2015).

Finally, it should be noted that PSGs upregulated during aging were enriched for terms related to inflammation that are also known to be a highly conserved hallmark of aging at the transcriptome level (Baumgart *et al*., 2014).

## Experimental procedures

### Genome‐scale identification of positively selected genes

The basis for this work were protein‐coding sequences (CDSs) of six *Nothobranchius* species (*N. furzeri*,* N. kadleci*,* N. kuhntae*,* N. pienaari*,* N. rachovii, and N. korthausae*) and *A. striatum* from transcriptome catalogs that were recently assembled and annotated (Reichwald *et al*., 2015) with FRAMA (Bens *et al*., 2016). The reads were adapter clipped with seqprep (https://github.com/jstjohn/SeqPrep) and quality trimmed with sickle (Joshi & Fass, 2011) before assembly [for more information about tissues, read numbers, filtered bases, etc., see Table S12 (Supporting information) or (Reichwald *et al*., 2015)]. CDSs from seven additional outgroups (*Xiphophorus maculatus*,* Poecilia formosa*,* Fundulus heteroclitus*,* Maylandia zebra*,* Pundamilia nyererei*,* Stegastes partitus*,* Oryzias latipes*) were obtained from NCBI RefSeq (14.12.15) and assigned to ortholog groups by the best bidirectional BLAST hit criterion Camacho *et al*. (2009) against *N. furzeri*.

For each N. furzeri CDS isoform, the most similar isoform of each other species was determined by pairwise comparison. To reduce the risk of aligning nonhomologous codons, these sequences were required to have additionally at least a similarity of 70% with *N. furzeri* and 50% with each other species on protein level. The selected isoforms in each ortholog group were aligned with PRANK (Loytynoja & Goldman, 2008), which is the alignment software of choice for positive selection analysis (Fletcher & Yang, 2010). The alignments were stringently filtered with GBLOCKS (Talavera & Castresana, 2007) to remove gaps and unreliable alignment columns around them that could produce false signals of positive selection (−b2 = total number of sequences in the alignment, b4 = 30, t=c). Then, for each alignment the branch‐site test of positive selection (Yang & Nielsen, 2002; Zhang *et al*., 2005) was applied: The respectively tested branch (LCA, FKK, or PR) was marked as ‘foreground’, and all other branches were marked as ‘background’. The program CODEML from the PAML (Yang, 2007) package was called separately for models M2a0 (model = 2, NSsites = 2; fix_omega = 1, omega = 1) and M2a (model = 2, NSsites = 2; fix_omega = 0, omega = 1) as described in the PAML User Guide (http://abacus.gene.ucl.ac.uk/software/pamlDOC.pdf). To calculate a *P*‐value, the chi‐square distribution with one degree of freedom was used to compare the likelihoods of both models: *P* = χ^2^ (2*(ln(likelihood(M2a))‐ln(likelihood(M2a0))),1).

Sites under positive selection were inferred by the Bayes empirical Bayes method (Yang *et al*., 2005) provided by CODEML. Sites that were predicted in a two amino acid frame next to a block which was deleted by GBLOCKS were removed and an adjusted *P*‐value calculated. For 9017, 12028, and 10976 genes, *P*‐values were calculated in the N‐, FKK‐ and PR‐branches, respectively (Table S5, Supporting information). We considered all genes with *P*‐values ≤ 0.05 as positively selected genes (PSGs).

Since high rates of false positive were detected in some automated genome‐scale scans for PSGs in the past (Mallick *et al*., 2009; Markova‐Raina & Petrov, 2011), we demanded our candidates to fulfill further strict filter criteria. Candidates were removed that had: (I) not at least one species from the sister branch of the tested branch in the alignment, for example, for the N‐branch the presence of A. striatum was demanded, (II) less than four species in the alignment, (III) remained only few columns (<60 or <66.67%) or N. furzeri codons (<60%) of the alignment after GBLOCKS filtering, (IV) disproportional dN/dS ratios (e.g., ≥100 in foreground branch, >1 in background branch, <0.85 in foreground branch) were calculated by CODEML or (V) had an unreliably high fraction of inferred positively selected sites (more than 20%). Finally, we inspected all candidates on the FKK‐ and PR‐branch manually as well as roughly ten percent of those on the LCA‐branch and removed ten additional candidates (<5%) in total.

### Phylogenetic tree

The phylogenetic tree that is needed for the analysis with CODEML was calculated based on the concatenated alignment of CDS isoforms of those 4865 genes with aligned isoforms from all species (Table S6, Supporting information). The final tree was the consensus of 1046 different trees created by splitting the alignment in fragments of 15 knt and calculating a tree for each fragment with DNAML from the PHYLIP (Felsenstein, 2005) package. All PSGs that were predicted with this globally estimated tree were again tested for positive selection with the same methods described above but with a tree that was estimated on the alignment of the respective gene. 65 of 75, 79 of 88, and 100 of 106 candidates were confirmed by this approach in the N‐, FKK‐, and N‐branch, respectively (candidates that could not be confirmed are marked in Tables S1–S3, Supporting information).

### Hypothesis‐driven GO enrichments

We determined potential enrichments for the GO categories mitochondrial ribosome (GO:0005761) and mitochondrial respiratory chain complex I (GO:0005747) with Fisher's exact test. The set of tested genes that could be converted to entrez IDs served as background, that is, 7523, 9416, and 8745 genes for the N‐, FKK‐, and PR‐branch, respectively (Table S7, Supporting information). As this was an hypothesis‐driven approach, the *P*‐values were not corrected for multiple testing.

### Mitochondrial biogenesis enrichment simulation

For each of three tested branches, we built two gene sets, a background gene set and a mitochondrial biogenesis gene set. The background gene sets consisted of the tested genes of the respective branch that could be converted to Entrez IDs (https://www.ncbi.nlm.nih.gov/Entrez), that is, 7523, 9416, and 8745 genes for the N‐, FKK‐, and PR‐branch, respectively (Table S7, Supporting information). To avoid biases due to expression differences between gene sets, we reduced the background sets to those genes within the 5–95% expression quantile of the union of PSGs across the three branches. This resulted in 6803, 8336, and 7882 genes, respectively (Tables S8 and S11, Supporting information). For the mitochondrial biogenesis gene sets, a union was built from the genes enlisted in the following five mitochondrial‐related GO terms (GO:0000959, 0032543, 0045333, 0033108, 0070584). This union encompassed 331 genes (Table S9, Supporting information). For each branch, the mitochondrial biogenesis gene set consisted of the genes from this union that were also present in the background of the respective branch, resulting in 221, 250, and 245 genes, respectively (Table S9, Supporting information). In each simulation, round drawings were done for each branch from the respective background set and as often as PSGs were identified in that branch and could be converted to Entrez IDs, that is, 65, 73, 89 times (Table S10, Supporting information), respectively. Our simulation was conservative in the way that we did not reduce the number of drawings in each branch to the 5–95% expression quantile of the PSGs, giving the simulation a higher chance to draw genes from mitochondrial biogenesis set than we had in reality. At the end of each simulation round, it was counted how many drawn genes were in the mitochondrial biogenesis gene set for each branch and, finally, the sum across the three branches was calculated. One million simulation rounds were done.

## Author's contributions

AS and MB performed the analysis; MP and AC supervised the work; and AS, MP, and AC wrote the manuscript.

## Funding

This work was supported by the Leibniz Association (SAW‐2012‐FLI) and the German Research Foundation (DFG: PL 173/8‐1) and a grant from Scuola Normale Superiore (CELLSNS2015).

## Conflict of interest

None declared.

## Supporting information


**Table S1** Results of positive selection analysis in the N‐branch, ranked by *P*‐value.
**Table S2** Results of positive selection analysis in the PR‐branch, ranked by *P*‐value.
**Table S3** Results of positive selection analysis in the FKK‐branch, ranked by *P*‐value.
**Table S4** Comparison of fold‐changes in expression of mitonuclear and mitochondrial biogenesis gene sets.
**Table S5** Overview of the tested genes.
**Table S6** List of genes used to construct the tree in Fig. 1.
**Table S7** List of background genes used for GO analysis.
**Table S8** List of genes used as background for the simulation experiment (5–95% expression quantile of the PSGs).
**Table S9** List of mitochondrial biogenesis genes within the background genes list (Table S7).
**Table S10** Entrez orthologs of PSGs.
**Table S11** Expression levels of all tested genes.
**Table S12** Statistics of read data for each library.
**Table S13** Complete list of *Nothobranchius* PSGs overlapping with PSGs in long‐lived mammals.
